# Hedonic and eudaimonic well-being for sustainable development in university students: personality traits or acceptance of change?

**DOI:** 10.3389/fpsyg.2023.1180995

**Published:** 2023-05-15

**Authors:** Annamaria Di Fabio, Letizia Palazzeschi, Antonia Bonfiglio, Alessio Gori, Andrea Svicher

**Affiliations:** ^1^Department of Education, Languages, Intercultures, Literatures and Psychology (Psychology Section), University of Florence, Florence, Italy; ^2^Department of Health Sciences (Psychology Section), University of Florence, Florence, Italy; ^3^THE-Tuscany Health Ecosystem Next Generation UE-NRRP, Department of Education, Languages, Intercultures, Literatures and Psychology (Psychology Section), University of Florence, Florence, Italy

**Keywords:** acceptance of change, hedonic well-being, eudemonic well-being, personality traits, sustainability, sustainable development, good health and well-being

## Abstract

**Introduction:**

The psychology of sustainability and sustainable development aims to contribute to the establishment of a culture of sustainability regarding the 2030 Agenda and its 17 sustainable development goals.

**Methods:**

In this framework, this study examined the associations between acceptance of change and well-being (hedonic and eudaimonic sides), controlling for the effects of personality traits, in 284 Italian university students.

**Results:**

Acceptance of change explained additional variance over personality traits regarding hedonic and eudaimonic well-being.

**Discussion:**

Acceptance of change could thus represent a promising well-being resource from the perspective of strength-based prevention, opening future perspectives to face the challenges of sustainable development, particularly concerning Goal 3 of the 2030 Agenda: “Good health and well-being.”

## Introduction

The 2030 Agenda of the United Nations has advanced 17 sustainable development goals (see Table 1) to promote sustainability worldwide. The psychology of sustainability and sustainable development (PSSD) (Di Fabio and Rosen, 2018, 2020; Rosen and Di Fabio, n.d.) is a current research area contributing to the transdisciplinary framework of sustainability science (Rosen, 2017), and it supports a preventive culture regarding the 2030 Agenda and its 17 sustainable development goals.

**Table 1:** The 17 sustainable development goals of the 2030 Agenda.

GOAL 1 No poverty
GOAL 2 Zero hunger
GOAL 3 Good health and well-being
GOAL 4 Quality education
GOAL 5 Gender equality
GOAL 6 Clean water and sanitation
GOAL 7 Affordable and clean energy
GOAL 8 Decent work and economic growth
GOAL 9 Industry, innovation and infrastructure
GOAL 10 Reduced inequality
GOAL 11 Sustainable cities and communities
GOAL 12 Responsible consumption and production
GOAL 13 Climate action
GOAL 14 Life below water
GOAL 15 Life on land
GOAL 16 Peace and justice strong institutions
GOAL 17 Partnerships to achieve the goal

Currently, we are facing enormous challenges in an even more turbulent scenario than that which appeared at the beginning of the 21st century (Blustein et al., 2019); it is impacting the labor market and is characterized by change and instability. This new scenario is accelerating and increasing in intensity: on the one hand are acceleration, change, and precariousness; on the other hand are pandemics, war, climatic changes, etc. To deal with these changeable and demanding new contexts, people must adapt incessantly to change, and strength is required to constructively cope with change (Di Fabio and Gori, 2016a). Individuals who consider change as a possibility to discover and develop have a higher probability of responding positively to the difficulties of the present scenario (Blustein et al., 2019), successfully facing threats and shifts and thus enhancing well-being (Di Fabio and Kenny, 2016).

### Theoretical background

The concept of acceptance of change (Di Fabio and Gori, 2016a) refers to the tendency to encompass change. It includes the following factors: predisposition to change—the perception of individuals that they might acquire something as a result of change by utilizing change to increase the quality of their lives; support for change—support is perceived to be received from other people in the face of changes; change seeking—behavior where a person pursues change; acquiring and retaining information as well as exhibiting a need to receive novel stimulation; a positive reaction to change as perceived by positive emotions resulting from changing; positively experiencing and benefiting from change. Cognitive flexibility is perceived as having the “ability to think about multiple concepts simultaneously, to change decisions if this is advantageous, and to change plans and routines easily” (Di Fabio and Gori, 2016a, p. 2).

Resistance to change (Oreg, 2003) has traditionally been studied in literature and is considered the dark side of change processes. With the introduction of the acceptance of change (Di Fabio and Gori, 2016a), a new positive preventive perspective was proposed concerning change processes based on promoting resources and not only on reducing dysfunctionalities (Di Fabio, 2017a). From this perspective, acceptance of change is conceptualized as a resource to constructively face changes, permitting individuals to find ways to deal successfully with challenges and promote their well-being (Di Fabio and Gori, 2016a). This perspective is in line with positive psychology (Seligman and Csikszentmihalyi, 2000; Seligman, 2002), which is focused on the study of well-being by considering human strengths instead of failures. In this framework, the acceptance of change represents a positive resource for individuals to cope with the complex challenges they can meet in their lives.

Occupational health psychology has emphasized the value of a positive health perspective by considering the relevance of promoting the health, well-being, flourishing, and optimal functioning of workers (Tetrick and Peiró, 2012). It is proposed (Di Fabio et al., 2020) that this positive approach is integrated with a strength-based prevention perspective (Di Fabio and Saklofske, 2021) for healthy organizations. The focus is on a primary preventive approach focused on building workers’ positive individual resources for enhancing both well-being and performance in organizations (Di Fabio et al., 2020), thus facilitating the achievement of the third goal of the 2030 Agenda, “Good health and well-being.”

According to this perspective, well-being has to be considered both from the hedonic (Kahneman et al., 1999) and eudaimonic perspectives (Ryff and Singer, 2008) as well as from strength-based prevention perspectives (Di Fabio and Saklofske, 2021) considering the crucial asset of constructing personal resources (Di Fabio and Kenny, 2021) to foster well-being. In this preventive framework, including a primary preventive perspective (Di Fabio and Kenny, 2021), the acceptance of change is conceived as a promising resource related to well-being, advancing the research related to determinants of well-being, personality factors, and personal and environmental resources (Ramaci et al., 2020; Bellini et al., 2022; De Giorgio et al., 2023).

In the literature, some constructs holding the same perspective of acceptance of change in organizations were studied in relation to well-being: readiness for change (Helfrich et al., 2018), commitment to change (Jing et al., 2014), and change culture (Quigley et al., 2022). Specifically, regarding the relationships between acceptance of change and well-being, acceptance of change was positively associated with both hedonic well-being (life satisfaction) and eudaimonic well-being (flourishing) in workers and students (Di Fabio and Gori, 2016a). Another study (Di Fabio et al., 2016) conducted on Italian workers reported positive correlations between acceptance of change and both life satisfaction and meaning in life. Furthermore, two further studies conducted on Italian workers indicated that acceptance of change was positively linked to job satisfaction (Di Fabio and Gori 2020; Gori and Topino, 2020). In this context, acceptance of change appears particularly promising in relation to “good health and well-being,” the third of the 17 SDGs of the 2030 Agenda of the United Nations. Therefore, acceptance of change emerges as a deeply embedded theme in the PSSD research area (Di Fabio, 2017a,b; Di Fabio and Rosen, 2018, 2020), which also highlights the importance of prevention.

Analyzing the literature, to the best of our knowledge, no research exists that has specifically studied the relationships between the acceptance of change construct (Di Fabio and Gori, 2016a) and well-being that also considers personality traits. Furthermore, concerning the acceptance of change, no studies have simultaneously considered the following aspects of hedonic well-being: positive affect, negative affect, and life satisfaction; and the same can be said for the following aspects of eudaimonic well-being: meaning in life and flourishing. Consequently, the following hypotheses are proposed: acceptance of change explains additional variance regarding positive affect (H1), negative affect (H2), life satisfaction (H3), meaning in life (H4), and flourishing (H5) beyond that accounted for by personality traits.

## Method

### Participants and procedure

A total of 284 university psychology students from the University of Florence (28.52% male and 71.48% female; mean age = 22.81 years, SD = 1.88) participated in the study. University students participated voluntarily in the study and were not compensated. They provided informed consent. Instruments were administered to groups by specialized personnel adhering to Italian privacy laws (DL-196/2003; EU 2016/679). The administration order of the measures was balanced to contain the presentation order effects. The study was approved by the Ethical Committee of the Integrated Psychodynamic Psychotherapy Institute (IPPI) (IPPI Ethical Committee Number 016/2022).

### Measures

#### We used The following measures.

*Big five questionnaire* (BFQ; Caprara et al., 1993), 132 items (1–5, from «*Absolutely false*» to «*Absolutely true*»), five factors: emotional stability (Alpha = 0.90), extraversion (Alpha = 0.81), conscientiousness (Alpha = 0.81), Openness (Alpha = 0.75), and Agreeableness (Alpha = 0.73).

*Acceptance of change scale* (ACS; Di Fabio and Gori, 2016a), 20 items (1–5, from «*Not at all* » to «A *great deal*»), five dimensions: predisposition to change (Alpha = 0.83), support for change (Alpha = 0.79), change seeking (Alpha = 0.80), positive reaction to change (Alpha = 0.75), and cognitive flexibility (Alpha = 0.72).

*Positive and negative affect schedule* (PANAS; Watson et al., 1988; Italian version Terraciano et al., 2003), 20 adjectives (1–5, from «*Very slightly or not at all*» to «*Extremely*»), PA (Alpha = 0.83), and NA (Alpha = 0.85).

*Satisfaction with life scale* (SWLS; Diener et al., 1985, Italian version, Di Fabio and Gori, 2016b): 5 items (1–7, from «*Strongly disagree*» to «*Strongly agree*») and Alpha coefficient: 0.85.

*Meaning in life measure* (MLM; Morgan and Farsides, 2009, Italian version Di Fabio, 2014): 23 items (1–7, from «*Strongly disagree*» to «*Strongly agree*»), five dimensions: exciting life, accomplished life, principled life, purposeful life, valued life, and alpha coefficient: 0.85 (total score).

*Flourishing scale* (FS; Diener et al., 2010, Italian version by Di Fabio, 2016): 8 items (1–7, from «*Strongly disagree*» to «*Strongly agree*») and Alpha coefficient: 0.88.

### Data analysis

Descriptive statistics, Pearson’s *r* correlations, and hierarchical regressions were calculated using the IBM SPSS Statistics software (version 28). We carried out hierarchical regressions with personality traits during the first step, acceptance of change dimensions during the second step, and alternated positive affect, negative affect, satisfaction with life, meaning in life, and flourishing as the dependent variables.

## Results

Table 2 presents the descriptive statistics for the study variables.

**Table 2:** Descriptive statistics for the study variables.

	*M*	SD	Skewness	Kurtosis
1. BFQ extraversion	75.32	9.20	−0.31	0.34
2. BFQ agreeableness	79.51	9.00	−0.08	0.73
3. BFQ conscientiousness	82.07	9.90	0.55	0.16
4. BFQ emotional stability	68.45	12.57	−0.33	0.39
5. BFQ openness	82.02	9.20	0.13	−0.31
6. ACS predisposition to change	12.49	2.61	−0.05	0.27
7. ACS support for change	14.28	2.98	−0.30	−0.15
8. ACS change seeking	10.05	2.83	0.38	−0.16
9. ACS positive reaction to change	13.21	2.42	0.20	0.08
10. ACS cognitive flexibility	13.95	2.63	−0.14	0.26
11. PANAS positive affect	35.33	5.21	0.01	−0.08
12. PANAS negative affect	22.43	8.40	0.80	0.41
13. SWLS satisfaction with life	23.48	6.38	−0.53	0.09
14. MLM meaning in life	115.31	16.66	0.08	−0.42
15. FS flourishing	42.68	7.70	−0.21	−0.60

*N* = 284.

Table 3 presents Pearson’s *r* correlations for the study variables.

**Table 3:** Correlations among BFQ, ACS, PANAS, SWLS, MLM, FS.

	1	2	3	4	5	6	7	8	9	10	11	12	13	14	15
1. BFQ extraversion	–														
2. BFQ agreeableness	0.16*	–													
3. BFQ conscientiousness	0.17**	0.13*	–												
4. BFQ emotional stability	0.18**	0.28**	0.14*	–											
5. BFQ openness	0.21**	0.44**	0.17**	0.17**	–										
6. ACS predisposition to change	0.34**	0.13*	0.06	0.31**	0.28**	–									
7. ACS support for change	0.12*	0.22**	0.01	0.23**	0.02	0.34**	–								
8. ACS change seeking	0.10	0.03	−0.16**	0.09	0.23**	0.40**	0.10	–							
9. ACS positive reaction to change	0.16**	0.16**	0.03	0.19**	0.19**	0.45**	0.27**	0.34**	–						
10. ACS cognitive flexibility	0.12*	0.22**	0.11*	0.10	0.17**	0.33**	0.27**	0.28**	0.37**	–					
11. PANAS positive affect	0.53**	0.07	0.23**	0.17**	0.23**	0.40**	0.33**	−0.11	0.23**	0.12*	–				
12. PANAS negative affect	−0.15*	−0.35**	−0.03	−0.39**	−0.24**	−0.05	−0.17**	0.14*	−0.09	−0.05	−0.14*	–			
13. SWLS satisfaction with life	0.32**	0.26**	0.10	0.27**	0.12	0.35**	0.43**	−0.02	0.04	0.14*	0.47**	−0.29**	–		
14. MLM meaning in life	0.44**	0.30**	0.26**	0.25**	0.36**	0.40**	0.16**	−0.26**	0.06	0.09	0.64**	−0.40**	0.63**	–	
15. FS flourishing	0.40**	0.31**	0.21**	0.22**	0.34**	0.41**	0.37**	−0.13*	0.09	0.07	0.58**	−0.36**	0.58**	0.77**	–

*N* = 284. * < 0.05, ** < 0.01.

Table 4 presents the results of the hierarchical regressions.

**Table 4:** Hierarchical regression: contribution of big five (BFQ) and ACS dimensions in relation to PANAS, SWLS, MLM, FS.

	PANAS PA	PANAS NA	SWLS	MLM	FS
	*β*	*β*	*β*	*β*	*β*
*Step 1*					
BFQ extraversion	0.46**	−0.07	0.28**	0.33**	0.31**
BFQ agreeableness	0.06	−0.24**	0.22**	0.14**	0.18**
BFQ conscientiousness	0.16**	−0.04	0.06	0.17**	0.12**
BFQ emotional stability	0.11**	−0.29**	0.18**	0.14**	0.10**
BFQ openness	0.12**	−0.08	0.08	0.18**	0.16**
*Step 2*					
ACS predisposition to change	0.14*	−0.13	0.23**	0.30**	0.31**
ACS support for change	0.27**	−0.08	0.32**	0.14*	0.29**
ACS change seeking	−0.00	0.20**	−0.08	−0.24**	−0.14*
ACS positive reaction to change	0.04	−0.06	0.05	0.01	0.14*
ACS cognitive flexibility	0.08	−0.01	0.16**	0.12	0.13
*R*^2^ *step 1*	0.32***	0.23***	0.19***	0.33***	0.28***
Δ*R*^2^ *step 2*	0.10***	0.05**	0.15***	0.12***	0.16***
*R*^2^ *total*	0.42***	0.28***	0.34***	0.45***	0.44***

*N* = 284; * < 0.05, ** < 0.01, ***p < 0.001.

Regarding positive affect, BFQ explained 32% of the variance, and the ACS dimensions explained 10%, for a total variance of 42%.

Regarding negative affect, BFQ explained 23% of the variance, and the ACS dimensions explained 5%, for a total variance of 28%.

Regarding satisfaction with life, BFQ explained 19% of the variance, and the ACS dimensions added 15%, for a total variance of 34%.

Concerning meaning in life, the BFQ explained 33% of the variance, and the ACS dimensions added 12%, for a total variance of 45%.

Concerning flourishing, BFQ explained 28% of the variance, and the ACS dimensions added 16%, for a total variance of 44%.

## Discussion

This study analyzed, for the first time, the associations between the acceptance of change construct (Di Fabio and Gori, 2016a) and both hedonic (PA, NA, SWLS) and eudaimonic well-being (MLM, FS), considering personality traits, in Italian university students. Our findings support this hypothesis.

Regarding hedonic well-being, the results confirmed the first hypothesis. Acceptance of change explained additional variance to the big five for positive affect. Particularly, regarding positive affect, positive significant relationships emerged with the predisposition to change dimension as well as with support for changing dimension. Aspects of acceptance of change relative to individuals’ perceptions of acquiring from change and applying changes to increase their quality of life as well as perceiving social support in coping with changes (Di Fabio and Gori, 2016a) are related to the propensity to experience positive emotions (Watson et al., 1988). These results highlighted that considering change as a positive challenge and perceiving support from others in facing change were associated with the positive affect experienced by the participants in this study.

The findings confirm the second hypothesis. Acceptance of change explained additional variance to the big five for negative affect. Particularly, negative affect indicated a significant direct relationship with the change-seeking dimension. This relationship is interesting, even if it may seem counterintuitive at first. In this study, a search for change was associated with the experience of negative affect, probably because the perception of looking for change and exhibiting a necessity for new stimuli (Di Fabio and Gori, 2016a) could be connected to encountering the world more negatively (Watson et al., 1988), and perhaps a need for change could emerge.

Thus, the third hypothesis was confirmed. Acceptance of change explained the additional variance to the big five for life satisfaction. Particularly, life satisfaction was positively associated with support for change, predisposition to change, and cognitive flexibility dimensions, in this order of importance. In this study, different aspects of acceptance of change were associated with the global satisfaction of an individual’s existence (Diener et al., 1985): the perception of support received by others in facing change, primarily the perception of being predisposed to change, and the perception of having the capacity to shift between various conceptions using adaptive cognitive strategies. A global positive evaluation of one’s life includes aspects of relational satisfaction (Diener et al., 1985) and thus appears to be associated with the perception of being supported by others in the face of changes. Moreover, being satisfied with one’s life includes aspects related to a predisposition to change regarding the perception of having opportunities to learn from change as well as the perception of being able to face the challenges of life (Diener et al., 1985). Life satisfaction, as a cognitive aspect of hedonic well-being regarding favorable evaluation of personal life rather than present feelings (Diener et al., 1985), was also connected to the cognitive flexibility of acceptance of change in this study. It is worth emphasizing that life satisfaction was the only aspect of well-being significantly associated with the cognitive flexibility dimension of acceptance of change in this study, probably because these two variables are more closely linked to cognitive processes. The findings of this study, thus, documented the relationships between acceptance of change and diverse facets of hedonic well-being, even after considering personality.

Regarding eudaimonic well-being, our results confirmed the fourth hypothesis. Acceptance of change explained additional variance to the big five for meaning in life. Meaning in life indicated significant positive relationships with the predisposition to change and support for change dimensions and a significant inverse relationship with the change-seeking dimension. In this study, a greater acknowledgment and awareness of meaningful and authentic goals (Morgan and Farsides, 2009) is positively related to different features of acceptance of change regarding the perception of being predisposed to change and being supported by others when facing changes. The findings emphasize the value of a positive attitude toward change concerning predisposition to change and support for change in eudaimonic well-being as authenticity and self-realization. The inverse relationship between the change-seeking dimension and meaning in life could highlight that the participants in this study, seeking new stimuli and probably experiencing a less meaningful life, could be pushed towards novelties.

Finally, the fifth hypothesis was confirmed. Acceptance of change explained the additional variance to the big five for flourishing. Particularly, flourishing indicated significant positive relationships with the predisposition to change, support for change, and positive reaction to change dimensions, whereas a significant inverse relationship emerged with the change-seeking dimension. It is possible to notice a more comprehensive form of eudaimonic well-being, namely, flourishing, defining it as the perception of psychological well-being concerning “relationships, self-esteem, purpose, and optimism” (Diener et al., 2010, p. 143) that resulted from the majority of the dimensions of acceptance of change, including also the positive reaction to change dimension. In this study, acceptance of change in terms of predisposition to change, support for change, and positive reaction to change seem to be relevant for achieving a form of eudaimonic well-being that permits flourishing, functioning optimally, and developing to the best of one’s possibilities (Diener et al., 2010). Furthermore, the link between flourishing and change seeking was inverse, indicating that in this study, when participants sought change, they appeared to experience less eudaimonic well-being regarding flourishing, just as the desire to change appears to be motivated by a desire to achieve greater overall eudaimonic well-being. Thus, acceptance of change emerged in this study regarding aspects of eudaimonic well-being concerning both meaning in life and flourishing.

Further reflections can be emphasized regarding the associations between acceptance of change and different forms of well-being. In this study, the contribution of acceptance of change was greater for the eudaimonic well-being aspect of flourishing, followed by satisfaction with life for hedonic well-being and meaning in life for eudaimonic well-being as the third aspect. Acceptance of change appears to be related to a great flourishing of eudaimonic well-being and, subsequently, be associated with a great cognitive reflection on global satisfaction with one’s own life (Diener et al., 1985) for hedonic well-being and is related to meaning in life (Morgan and Farsides, 2009) for eudaimonic well-being. In this study, the perception of accepting change seems to be relevant, particularly in forms of eudaimonic well-being as functioning optimally, emphasizing self-expression and self-realization (Diener et al., 2010), and adherence to authentic meanings and values (Morgan and Farsides, 2009), but also with hedonic well-being, especially regarding life satisfaction, suggesting that being open to changes could be linked to various types of well-being.

Despite the results obtained, this study has some limitations that must be addressed. First, a limitation relative to the participants is that students in psychology at the University of Florence were predominantly female. Even if this composition of the group of participants tends to reflect the distribution of gender among psychology students, it remains a limitation of this study. Future studies should be conducted considering a better balance between males and females, as well as the inclusion of students from various disciplines and from other universities in Italy. Future studies could extend this study to different international contexts. A further limitation is that the study used self-reported measures. The cross-sectional design constitutes another limitation, suggesting a longitudinal approach for future research. Additionally, future research could consider studying these relationships in students attending high school as well as in other targets, such as workers. With this latter target, future studies could also investigate the acceptance of change regarding other specific aspects of well-being at work, such as job satisfaction (Judge et al., 1998) and work meaning (Steger et al., 2012).

## Conclusion

If these results are replicated, new perspectives on intervention can be opened. The current complex, unstable, and detonating scenario (Blustein et al., 2019) is calling for strength to cope with change in a constructive manner, and to successfully face transitions and adversities, so that the well-being of individuals is not threatened. In this scenario, acceptance of change emerges as a promising resource. In fact, acceptance of change is amenable to training, contrary to personality traits, which are generally stable (Costa and McCrae, 1992). Thus, helping individuals face the transforming and mutable environments of the current century effectively (Di Fabio and Gori, 2016a) could be a resource for enhancing their well-being. According to strength-based perspectives, especially in a primary preventive approach (Di Fabio and Kenny, 2021), acceptance of change could be configured as a promising resource to respond to the challenges connected in particular to the third sustainable development goal, “Good health and well-being” (SDG 3). Furthermore, from these perspectives, early preventive actions on acceptance of change for university students could also address the challenges of decent education (Duffy et al., 2022) and decent work (Duffy et al., 2017; Di Fabio and Kenny, 2019; Svicher et al., 2022). Improving resources for change in young people as future workers in organizations (Di Fabio and Blustein, 2016) could promote decent work as the eighth sustainable development goal (SDG8). Early preventive actions enhancing acceptance of change could also better deal with the challenges relative to all other sustainable development goals (SDGs) for the promotion and establishment of a culture of sustainability and sustainable development.

## Data availability statement

The raw data supporting the conclusions of this article will be made available by the authors, without undue reservation.

## Ethics statement

The studies involving human participants were reviewed and approved by Integrated Psychodynamic Psychotherapy Institute (IPPI). IPPI Ethical Committee Number 016/2022. The patients/participants provided their written informed consent to participate in this study.

## Author contributions

ADF conceptualized the paper. ADF, LP, AB, AG, and AS contributed to the data collection. LP ran statistical analyses. ADF and LP wrote the first draft of the paper. ADF, AS, and AG edited and wrote the final draft of the paper. All authors contributed to the article and approved the submitted version.

## Conflict of interest

The authors declare that the research was conducted in the absence of any commercial or financial relationships that could be construed as a potential conflict of interest.

## Publisher’s note

All claims expressed in this article are solely those of the authors and do not necessarily represent those of their affiliated organizations, or those of the publisher, the editors and the reviewers. Any product that may be evaluated in this article, or claim that may be made by its manufacturer, is not guaranteed or endorsed by the publisher.
